# Facilitators of and barriers to gastric cancer and precursor diagnosis among South Texas residents: Social determinants of health

**DOI:** 10.1002/cam4.7002

**Published:** 2024-03-20

**Authors:** Dorothy Long Parma, Erin P. Finley, Roman Fernandez, Jonathan A. L. Gelfond, Amelie G. Ramirez

**Affiliations:** ^1^ Department of Population Health Sciences, Long School of Medicine The University of Texas Health Science Center at San Antonio San Antonio Texas USA; ^2^ Department of Medicine and Psychiatry and Behavioral Sciences, Long School of Medicine The University of Texas Health Science Center at San Antonio San Antonio Texas USA; ^3^ Center for Healthcare Innovation, Implementation and Policy Virginia Greater Los Angeles Healthcare System Los Angeles California USA

**Keywords:** gastric cancer, *Helicobacter pylori*, Latinos/Hispanics, social determinants of health

## Abstract

**Background:**

Latinos/Hispanics are at higher risk for developing gastric cancer (GC) compared with non‐Hispanic whites, and social determinants of health (SDoH) are thought to contribute.

**Aims/Materials and Methods:**

This study addressed SDoH and their interactions contributing to disparities in the testing and treatment of Helicobacter pylori (HP) infection and diagnosis of GC and its known precursors, among Latinos/Hispanics relative to non‐Latinos at two affiliated but independent health systems in San Antonio, Texas, using a mixed methods approach.

**Results:**

Secondary data abstraction and analysis showed that GCs represented 2.6% (*n* = 600) of our population. Men and older individuals were at higher GC risk. Individuals with military insurance were 2.7 times as likely to be diagnosed as private insurance. Latinos/Hispanics had significantly (24%) higher GC risk than Whites. Poverty and lack of insurance contributed to GC risk among the minorities classified as other (Asians, Native Americans, Multiracial; all *p* < 0.01). All SDoH were associated with H. pylori infection (*p* < 0.001). Qualitative analysis of patient and provider interviews showed providers reporting insurance as a major care barrier; patients reported appointment delays, and lack of clinic staff. Providers universally agreed treatment of H. pylori was necessary, but disagreed on its prevalence. Patients did not report discussing H. pylori or its cancer risk with providers.

**Discussion/Conclusion:**

These data indicate the importance of considering SDoH in diagnosis and treatment of GC and its precursors, and educating providers and patients on H. pylori risks for GC.

## 1 INTRODUCTION

Compared to non‐Hispanic whites (NHWs), Latinos/Hispanics have higher risk of developing multiple cancers, including gastric cancer (GC).^1^ GC is the third‐leading cause of cancer death worldwide^2^ and was the sixth‐ and eighth‐leading cause for Texas men and women, respectively, in 2012–2016.^3^ Mortality rates were twice as high for Latino/Hispanic than NHW men and 2.4 times higher for Latino/Hispanic than NHW women. GC incidence has been increasing in persons aged <50 years and Latino/Hispanic women.^2^, ^4^ In 2018, the probability of developing invasive GC at any age was twice as high in Latino/Hispanic versus NHW men (1.6, or 1 in 64 vs. 0.8., 1 in 119), and three times higher in Latinas/Hispanics versus NHW women (1.2, 1 in 85 vs. 0.4, 1 in 227).^3^ Moreover, Latinos/Hispanics are diagnosed at younger ages and more advanced stages compared to other ethnic groups,^3^, ^5^, ^6^ which may contribute to higher mortality rates.^7^ Gastric adenocarcinomas (GCAs) account for approximately 90% of all GCs.^8^

Latinos/Hispanics have higher GC rates in the distal stomach,^9^ which has strong correlations with *Helicobacter pylori (H. pylori*) infection.^10^, ^11^, ^12^ *H. pylori* infection accounts for 90% of GC cases, and is considered the primary GC risk factor (RF).^13^ *H. pylori* influences several key elements in the Correa cascade of gastric carcinogenesis, including known GC precursors atrophic gastritis and intestinal metaplasia (IM).^14^, ^15^ IM is highly prevalent in U.S. Latinos/Hispanics; its location, predominantly in the distal stomach is consistent with that of the majority of Latino/Hispanic GC cases.^16^

The American College of Gastroenterology treatment guidelines for *H. pylori* recommend bismuth‐based quadruple therapy with clarithromycin, amoxicillin, and metronidazole.^17^ However, many practitioners still follow older guidance of triple therapy for *H. pylori*. This could be problematic in areas with high antibiotic resistance to clarithromycin and macrolides.

Latinos/Hispanics are disproportionately vulnerable to cancer due to the multiple social determinants of health (SDoH) including increased poverty, decreased education and low or no insurance.^3^ In South Texas (STX), a 38‐county, majority Latino/Hispanic (nearly 70%)^18^ region encompassing San Antonio south to the Lower Rio Grande Valley along the Texas‐Mexico border, about 22% of residents on average had an income below 150% of the federal poverty line in 2018 (range 7.3%–35%), compared with the U.S. national average of 12%.^19^ For the period 2007–2010, an estimated 30% of STX residents were uninsured compared to 23% in the rest of Texas and 15% nationwide. Latinos had the highest uninsured rate (40%).^20^ SDoH have contributed to racial‐ethnic disparities in GC incidence. A study of the National Cancer Institute (NCI) Surveillance, Epidemiology and End Results (SEER) program from 2000 to 2014 found a 2.8‐fold increased incidence of non‐cardia GC in Latinos/Hispanics and blacks. Incidence increased with decreasing neighborhood socioeconomic status (nSES) for both ethnic groups.^21^

In this paper, we describe concurrent mixed‐method research to investigate factors, including SDoH and their interactions, that contribute to disparities among Latinos/Hispanics relative to non‐Latinos, in diagnosis of *H. pylori* infection, GC, and known precursors. Findings draw upon secondary data collection/analysis of electronic health records (EHR) from two affiliated but independent health systems (quantitative); and semi‐structured interviews of providers and a convenience sample of eligible patients in gastroenterology and oncology clinics on their experiences of facilitators of and barriers to care for these disorders (qualitative).

## 2 MATERIALS AND METHODS

This study was approved by the Institutional Review Board of the University of Texas Health Science Center at San Antonio, 2019‐387E and 2019‐0534H. The study employed a concurrent mixed‐methods approach to collect and analyze quantitative and qualitative data to better understand the full scope of access barriers leading to health disparities in GC preventive care. The quantitative data characterizes the level of disparities in access, while the qualitative data invites multilevel perspectives on causes of these differences. We collected data using both methods in parallel, and integrated and reported on them contiguously, that is, in separate sections of the Results before combining them in the Discussion.^22^ This will lay the groundwork for future studies and interventions aimed at improving GC preventive care.

### 2.1 Quantitative

#### 2.1.1 Study cohort variables

Variables of interest were extracted from structured data in the Clinical Research Informatics (CRI) data warehouse on all patients with gastric disorders (*H. pylori* infection, gastritis, gastric ulcer, atrophic gastritis, IM, and GCA, gastric lymphoma and gastric MALT lymphoma) diagnosed 2007–2020. Structured data was examined using regular expression of coding strings, for example, billing ICD9/10 diagnostic codes, key words, and Logical Observation Identifiers Names and Codes (LOINC).^23^ Median income and educational attainment was obtained from the 2016 American Community Survey 5‐year Summary linked to patients based on their addresses; addresses were mapped to census block groups or zip codes and median income binned for the census group to de‐identify it for researcher use. Zip code data was used to identify median income via the U.S. Census,^24^ as well as estimates of geographical RF distribution.

#### 2.1.2 Quantitative analysis

Descriptive statistics were performed. Student's *t*‐test was used to examine relationships between Latinos/Hispanics and NHWs for continuous variables, and chi‐square test for categorical variables. Logistic regression models were used to examine differences between racial/ethnic groups with the outcomes of GC (vs. not) and other gastric diagnoses (atrophic gastritis, ulcer, gastritis, *H.pylori*, IM) controlling for demographics (age, gender) and socioeconomic covariates (patient‐level payer status and the following geocoded variables: minority population, high school education, median income, unemployment, uninsured status, and poverty levels). The geocoded variables were transformed into quartiles to improve interpretation. Named gastric diagnoses were evaluated individually. The interactions between socioeconomic covariates and race/ethnicities were tested to compare the effects of socioeconomic status within racial/ethnic strata. Time period was also adjusted for as a categorical covariate that represented 2‐year intervals from 2007 to 2020. This approach provided estimation of odds ratios and 95% confidence intervals. All testing was two‐sided at the significance level 0.05.

### 2.2 Qualitative

#### 2.2.1 Interview content and preparation

We developed semi‐structured interview guides for both providers and patients based in the Practical, Robust Implementation and Sustainability Model (PRISM) to ensure interviews included questions to inform understanding of key needs, barriers and facilitators at organizational, provider, and patient levels (please see Figures S1 and S2 for full interview guides).^25^ The provider interview focused on: perceptions of *H. pylori* infection's impact on their patients' quality of life; perceived diagnosis rates of *H. pylori* and related gastric disorders; knowledge of and attitudes toward current practice guidelines^13^; barriers to treatment initiation, completion and follow‐up; and perceived effectiveness of testing, treatment and follow‐up protocols. The patient interview focused on: understanding of the diagnosis and/or treatment plan; family history of *H. pylori* infection, gastric disorders and GC; barriers to treatment initiation, completion and follow‐up; and satisfaction with received care. We targeted the patient interview to a 6th grade reading level, translated it into Spanish, and had it vetted by bilingual IHPR and UT Health faculty and staff experienced in qualitative research; interviews took 30–60 min to complete. Interviewers were bilingual and trained in interviewing techniques to most effectively elicit forthright responses.

#### 2.2.2 Recruitment: Providers

We contacted UHS and UTHealth providers in Primary Care, Gastroenterology and Oncology with an introductory email explaining the study and requesting participation. Additional contact methods (email or telephone follow‐up) followed as needed to assess interest, answer questions, and schedule interviews; we asked all participants' permission to audio record interviews. We interviewed four oncologists, six gastroenterologists, and eight primary care physicians at both UHS and UTHealth. Responses were de‐identified to protect confidentiality. The PI conducted all provider interviews.

#### 2.2.3 Recruitment: Patients

Eight oncology patients and seven gastroenterology patients were interviewed from convenience samples of patients scheduled to be seen at oncology clinics or for EGD procedures. Interviews were conducted on‐site in‐person or via telephone at the participant's convenience. Patients were compensated for their time. The PI and a bilingual individual with an MPH conducted all patient interviews.

All responses were de‐identified to protect confidentiality; all interviews were audio recorded with participants' permission. All participants were provided with an information sheet explaining the purpose of the interview and the overall study.

#### 2.2.4 Qualitative data analysis

All interviews were recorded, transcribed, and analyzed for common themes using rapid qualitative approaches, specifically matrix techniques.^26^, ^27^ We defined a preliminary set of analytic themes to capture barriers and facilitators to care and other relevant factors, consistent with PRISM. We created templates to summarize theme content and then further condensed them into matrices by theme and participant. These were reviewed and compared within and across participants by two independent research staff. Reporting of results was conducted according to consolidated criteria for reporting qualitative studies (COREQ).^28^

## 3 RESULTS

### 3.1 Quantitative

#### 3.1.1 Diagnoses

The breakdown of diagnoses in our EHR‐based sample was as follows: *H pylori* infection 36%; gastritis 19%; gastric ulcer 14%; chronic/atrophic gastritis 9.4%; IM 0.5%; GCA 2.3%; gastric MALT lymphoma 0.2%. Of all patients, 55% were tested for *H. pylori*, and of these 46% tested positive.

#### 3.1.2 Demographic characteristics and gastric cancer risk

Table 1 shows demographic characteristics and GC risk of the EHR‐based sample (diagnoses 2007–2020). Over half of GC patients were Latino (Hispanic in Table 1) and male, and about a quarter had either Medicare or private insurance. Logistic regression adjusting for age and gender showed that Latinos had increased odds of GC diagnosis relative to whites (OR 1.24; 95% CI 1.02, 1.51; *p* = 0.031). Older age and male gender (*p* < 0.001; data not shown), and those with military insurance (insurance provided to military veterans) (OR = 2.67; CI 1.19, 5.40; *p* = 0.01) had increased odds of GC relative to younger age, female gender, and private insurance, respectively. Most year intervals were positively associated with GC (*p* < 0.001). In addition, low median income (4th quartile OR = 1.3; 95% CI 1.01, 1.69; *p* = 0.045) was also associated.

**TABLE 1:** Sample characteristics, social determinants, and gastric cancer risk.

Characteristic	Demographics		Logistic regression		
	0, *N* = 22,720^a^	1, *N* = 600^a^	OR	95% CI	*p*‐Value
Age	47 (35, 57)	62 (52, 70)	—	—	—
Sex					
Female	12,600 (66%)	258 (45%)	—	—	—
Male	6561 (34%)	318 (55%)	—	—	—
Race_Ethnicity_V2					
White	5830 (32%)	169 (31%)	—	—	—
Asian	527 (2.8%)	11 (2.0%)	0.76	0.38, 1.36	0.4
Black	1232 (6.7%)	37 (6.8%)	1.22	0.83, 1.74	0.3
Hispanic	10,025 (54%)	309 (57%)	1.24	1.02, 1.51	0.031
Other	885 (4.8%)	16 (3.0%)	0.79	0.45, 1.30	0.4
Payer class revised					
Private	3797 (17%)	140 (23%)	—	—	—
Carelink	9153 (40%)	116 (19%)	0.45	0.35, 0.58	<0.001
Government	1676 (7.4%)	21 (3.5%)	0.51	0.31, 0.80	0.005
Medicaid	1454 (6.4%)	51 (8.5%)	1.26	0.89, 1.75	0.2
Medicare	2356 (10%)	171 (28%)	0.88	0.68, 1.13	0.3
Medicare + Medicaid	72 (0.3%)	5 (0.8%)	0.57	0.14, 1.60	0.4
Military	102 (0.4%)	9 (1.5%)	2.67	1.19, 5.40	0.01
Missing	844 (3.7%)	45 (7.5%)	1.17	0.81, 1.66	0.4
Other	1188 (5.2%)	11 (1.8%)	0.31	0.15, 0.58	<0.001
Self‐Pay	2078 (9.1%)	31 (5.2%)	0.56	0.36, 0.86	0.011
Year_Interval					
2006–2008	4220 (19%)	14 (2.3%)	—	—	—
2008–2010	3890 (17%)	27 (4.5%)	1.55	0.82, 3.05	0.2
2010–2012	4200 (18%)	51 (8.5%)	2.3	1.30, 4.33	0.006
2012–2014	4123 (18%)	95 (16%)	4.32	2.54, 7.95	<0.001
2014–2016	2557 (11%)	141 (24%)	8.48	5.04, 15.5	<0.001
2016–2018	2591 (11%)	178 (30%)	10.4	6.19, 18.8	<0.001
2018–2020	1139 (5.0%)	94 (16%)	11.7	6.82, 21.7	<0.001
MINORITYPOP_2016_5Y_QUARTILE					
Q1	4995 (23%)	183 (32%)	—	—	—
Q2	4807 (22%)	95 (17%)	0.66	0.50, 0.85	0.002
Q3	4733 (21%)	101 (18%)	0.73	0.56, 0.94	0.016
Q4	7619 (34%)	185 (33%)	0.75	0.60, 0.93	0.01
NOHS_2016_5Y_QUARTILE					
Q1	3070 (14%)	87 (15%)	—	—	—
Q2	7814 (35%)	193 (34%)	0.97	0.75, 1.28	0.8
Q3	5666 (26%)	136 (24%)	0.93	0.70, 1.24	0.6
Q4	5604 (25%)	148 (26%)	0.97	0.73, 1.28	0.8
BAORHIGHER_2016_5Y_QUARTILE					
Q1	4617 (21%)	116 (21%)	—	—	—
Q2	4448 (20%)	101 (18%)	0.92	0.69, 1.22	0.6
Q3	6678 (30%)	171 (30%)	1.08	0.84, 1.39	0.5
Q4	6411 (29%)	176 (31%)	1.04	0.82, 1.34	0.7
MEDINCOME_2016_5Y_QUARTILE					
Q1	5200 (24%)	110 (20%)	—	—	—
Q2	5150 (24%)	125 (23%)	1.23	0.93, 1.61	0.14
Q3	5733 (26%)	152 (28%)	1.28	0.99, 1.67	0.061
Q4	5594 (26%)	162 (30%)	1.3	1.01, 1.69	0.045
UNEMPLOYED_2016_5Y_QUARTILE					
Q1	4976 (22%)	144 (26%)	—	—	—
Q2	5540 (25%)	159 (28%)	0.95	0.75, 1.20	0.7
Q3	5126 (23%)	119 (21%)	0.76	0.58, 0.98	0.034
Q4	6512 (29%)	142 (25%)	0.75	0.59, 0.96	0.02
UNINSURED_2016_5Y_QUARTILE					
Q1	4841 (22%)	119 (21%)	—	—	—
Q2	4235 (19%)	105 (19%)	1.06	0.81, 1.40	0.7
Q3	5206 (23%)	139 (25%)	1.16	0.90, 1.50	0.3
Q4	7872 (36%)	201 (36%)	1.09	0.86, 1.39	0.5
POOR_2016_5Y_QUARTILE					
Q1	2623 (12%)	77 (14%)	—	—	—
Q2	6032 (27%)	172 (30%)	1.03	0.77, 1.37	0.9
Q3	7504 (34%)	181 (32%)	0.93	0.70, 1.24	0.6
Q4	5995 (27%)	134 (24%)	0.85	0.63, 1.15	0.3
NEARPOOR_2016_5Y_QUARTILE					
Q1	3697 (17%)	96 (17%)	—	—	—
Q2	6233 (28%)	176 (31%)	1.24	0.96, 1.62	0.11
Q3	4603 (21%)	124 (22%)	1.24	0.94, 1.65	0.13
Q4	7621 (34%)	168 (30%)	0.95	0.73, 1.24	0.7
BELOWPOVERTY_2016_5Y_QUARTILE					
Q2	9532 (43%)	237 (42%)	—	—	—
Q3	5266 (24%)	158 (28%)	1.31	1.05, 1.62	0.014
Q4	7356 (33%)	169 (30%)	1.06	0.86, 1.30	0.6

^a^ Median (IQR); *n* (%).

Abbreviations: CI, confidence interval; OR, odds ratio; adjusted for age and sex.

#### 3.1.3 Social determinants of health and gastric cancer risk

Table 2 describes significantly increased GC risk due to interactions between race and ethnicity and socioeconomic factors derived from the American Community Survey of the year indicated. Results were reported at the census block level and adjusted by age, sex, and time. Poverty (OR = 1.46; CI 1.06, 2.03, *p* = 0.012) and lack of insurance (OR = 1.39; CI 1.06, 1.82, *p* = 0.017) contributed to increased GC risk for patients identified as other (including Asians, American Indian/Alaska Natives and Multi‐racial) when compared to whites.

**TABLE 2:** Race/ethnicity and SDoH interaction models.

Characteristic	OR	95% CI^a^	*p*‐value
Race_Ethnicity * MEDINCOME			
Black * MEDINCOME	1	1.00, 1.00	>0.9
Hispanic * MEDINCOME	1	1.00, 1.00	0.6
Other * MEDINCOME	1	1.00, 1.00	0.5
Race_Ethnicity * MINORITYPOP_QUARTILE			
Black * MINORITYPOP_QUARTILE	1.18	0.84, 1.69	0.3
Hispanic * MINORITYPOP_QUARTILE	1.09	0.91, 1.31	0.3
Other * MINORITYPOP_QUARTILE	1.19	0.89, 1.57	0.2
Race_Ethnicity * NOHS_QUARTILE			
Black * NOHS_QUARTILE	1.08	0.71, 1.64	0.7
Hispanic * NOHS_QUARTILE	1.11	0.90, 1.37	0.3
Other * NOHS_QUARTILE	1.24	0.90, 1.71	0.2
Race_Ethnicity * BAORHIGHER_QUARTILE			
Black * BAORHIGHER_QUARTILE	1.01	0.69, 1.48	>0.9
Hispanic * BAORHIGHER_QUARTILE	1.02	0.84, 1.23	0.9
Other * BAORHIGHER_QUARTILE	0.82	0.61, 1.10	0.2
Race_Ethnicity * MEDINCOME_QUARTILE			
Black * MEDINCOME_QUARTILE	0.88	0.63, 1.23	0.5
Hispanic * MEDINCOME_QUARTILE	0.97	0.80, 1.17	0.7
Other * MEDINCOME_QUARTILE	0.91	0.68, 1.23	0.5
Race_Ethnicity * UNEMPLOYED_QUARTILE			
Black * UNEMPLOYED_QUARTILE	1.06	0.75, 1.49	0.7
Hispanic * UNEMPLOYED_QUARTILE	1	0.83, 1.20	>0.9
Other * UNEMPLOYED_QUARTILE	1	0.74, 1.35	>0.9
Race_Ethnicity * UNINSURED_QUARTILE			
Black * UNINSURED_QUARTILE	1	0.71, 1.42	>0.9
Hispanic * UNINSURED_QUARTILE	1.08	0.91, 1.30	0.4
Other * UNINSURED_QUARTILE	1.39	1.06, 1.82	0.017
Race_Ethnicity * POOR_QUARTILE			
Black * POOR_QUARTILE	1.09	0.73, 1.62	0.7
Hispanic * POOR_QUARTILE	1.06	0.86, 1.30	0.6
Other * POOR_QUARTILE	1.46	1.06, 2.03	0.022
Race_Ethnicity * NEARPOOR_QUARTILE			
Black * NEARPOOR_QUARTILE	1.32	0.92, 1.91	0.14
Hispanic * NEARPOOR_QUARTILE	0.97	0.81, 1.17	0.8
Other * NEARPOOR_QUARTILE	1.52	1.14, 2.03	0.004
Race_Ethnicity * BELOWPOVERTY_QUARTILE			
Black * BELOWPOVERTY_QUARTILE	1.05	0.67, 1.66	0.8
Hispanic * BELOWPOVERTY_QUARTILE	0.97	0.76, 1.23	0.8
Other * BELOWPOVERTY_QUARTILE	1.02	0.70, 1.47	>0.9

Abbreviations: BAORHIGHER_QUARTILE, Proportion with bachelors degree or higher; BELOWPOVERTY_QUARTILE, Proportion families below poverty; CI, confidence interval; MEDINCOME_QUARTILE, Median income quartile; MINORITYPOP_QUARTILE, Minority population quartile; NEARPOOR_QUARTILE, Proportion near poverty: 100 and 125 percent of the poverty thresholds and referred to this group as near poor; NOHS_QUARTILE, Proportion without high school quartile; OR, odds ratio; POOR_QUARTILE, Proportion of population below poverty quartile; UNEMPLOYED_QUARTILE, Proportion unemployed quartile; UNINSURED_QUARTILE, Proportion uninsured quartile.

#### 3.1.4 Social determinants of health and risk of other gastric disorders

Additional bivariate analyses from the dataset of other gastric disorders (see Table S1) showed: Hispanic/Latino (OR = 1.41; CI 1.32, 1.51), Carelink safety net insurance (OR = 2.05; CI 1.88, 2.24), Medicaid (OR = 1.4; CI 1.22, 1.61), self‐pay insurance (OR = 1.19; CI 1.04, 1.36); minority population (OR = 1.67 for the 4th quartile; CI 1.54, 1.81), no high school (OR = 1.7; CI 1.54, 1.88), uninsured (OR = 1.52; CI 1.40, 1.64), unemployed (OR = 1.18; CI 1.09, 1.28), poverty (OR = 1.49; CI 1.34, 1.65) variables were all positively associated with *H. pylori* infection (all *p* < 0.001). Hispanic/Latino (OR = 1.18; CI 1.08, 1.28) and self‐pay insurance (OR = 1.76; CI 1.54, 2.01) were also associated with gastritis diagnosis (*p* < 0.001); Medicare + Medicaid (OR = 2.75; CI 1.66, 4.54) with gastric ulcer (*p* < 0.001); minority population (OR = 1.22 for the 4th quartile; CI 1.07, 1.38), no high school (OR = 1.25; CI 1.07, 1.47) and uninsured (OR = 1.16; CI 1.02, 1.32) with atrophic gastritis (all *p* < 0.01); low median income with IM (OR = 4.14; CI 2.35, 7.89; *p* < 0.001) and gastric ulcer (OR = 1.18; CI 1.05, 1.33; *p* = 0.006 for the 3rd quartile). Interaction analyses showed that all minorities with low median income were associated with *H. pylori* infection (*p* < 0.004; Table S2).

### 3.2 Qualitative

#### 3.2.1 Demographics

Demographic breakdown of patients interviewed was as follows: median age 58 years; 50% female; 8 of 15 Latino/Hispanic; majority white; 8 of 15 1st generation (i.e., immigrated from Mexico or other Latin American country); median time in U.S. if born in another country 15 years.

#### 3.2.2 Provider perspectives

Qualitative analyses of provider interviews revealed that providers were split on whether they viewed H. pylori prevalence as low or high in their patient population, but universally agreed they would treat it if diagnosed; however, only GIs and 2 PCPs reported preferring the recommended quadruple therapy. On being probed on guideline use, only this group identified the American Gastroenterology Association (AGA) guidelines as a key resource. All preferred the stool antigen test for diagnosis (consistent with guidelines) and cited symptoms (e.g., persistent epigastric pain, history of H. pylori infection with unclear eradication) as guides or “red flags” for the decision to test for H. pylori. Only site B providers, who serve low socioeconomic patients, and oncologists mentioned insurance and language as barriers to care, but six providers across specialties mentioned insurance policies as a needed improvement: specifically, they felt that patients could benefit from having more access to financial counselors, fewer requirement for prior authorization for drugs, and fewer changes to formularies, and expressed concerns some drugs in the AGA‐recommended treatment cocktail were not covered. Providers were also split on whether they perceived the prevalence of antibiotic resistance as high or unknown; they cited varied perspectives on the source of resistance, from providers using incorrect regimens to problems with patient compliance, as well as system‐level factors leading to patients not receiving the complete regimen. Four of six GI providers felt that referring providers were unaware or variably aware of H. pylori management guidelines. Nine primary care providers and GIs mentioned provider education as a needed improvement as well, particularly on AGA guidelines, appropriate H. pylori treatment regimens and eradication procedures, and when to refer to GI.

#### 3.2.3 Patient perspectives

About half of patients reported variable or poor health (due to chemotherapy for oncology patients), but relatively few reported overall barriers to accessing care. A few patients mentioned delays in obtaining appointments, and the lack of enough providers and other clinic staff. Most oncology patients were on chemotherapy, while GI patients were on reflux medication. Most patients identified abdominal and throat symptoms as part of their gastric disorder and had undergone endoscopy for diagnosis. They also cited information sources on their disorder, including materials from their doctor and the internet. Patients varied in whether they recalled having any discussion of *H pylori* (HP) by setting of care. H. pylori was reported as not discussed with providers in seven of eight oncology patients compared to two of seven GI, and the majority received no information on its risk for cancer. Of those who discussed H. pylori, three mentioned the “stool test.” All patients stated completing treatment was important; oncology patients cited survival while GI patients focused on symptom control. However, 7 of 15 cited side effects as a barrier. All patients were satisfied with their care overall. A few patients and providers both pointed to the need for additional clinic staff. When asked what advice they would give family members in a similar situation, six patients indicated they would advise their families to consult a doctor sooner for symptoms.

## 4 DISCUSSION

In the context of increased risk for GC and precursor conditions among Latinos, we set out to examine relationships between SDoH and risk of GC and its precancerous conditions, including H. pylori infection, using a mixed‐methods approach. We collected data using both methods in parallel, and integrated and reported on them contiguously, that is, in separate sections of the Results before combining them in the Discussion.^22^ First, we found that H. pylori diagnosis prevalence in this STX population was higher than national data (36% vs. 30%)^29^ and that seroprevalence was approaching that of a study conducted among a Mexican‐American population in Central Texas (46% vs. 57%).^30^ This also matches half of our interviewed providers' estimates of high *H. pylori* seroprevalence when testing levels are high (55% tested in this population, 46% H. pylori positive). However, a minority of providers preferred guideline‐recommended quadruple therapy for H. pylori, leading to a disconnect between testing and treatment practices. We also found that Latino/Hispanic ethnicity, older age and male gender, and military insurance were independently associated with increased risk of GC. When we studied interactions of ethnicity with SDoH, we found that lack of insurance and poverty increased minorities' risk for GC. These findings agree with a SEER 2000–2014 study that showed higher incidence rate ratios of non‐cardia GC among minorities with decreasing nSES,^21^ and a study that looked at nativity in Texas Latinos/Hispanics with GC.^31^ Insurance is a common factor in these findings and was also mentioned as a barrier to care by some providers, who also mentioned a need for provider education. These data indicate an opportunity to educate providers on H. pylori prevalence and recommended treatment, and the need to improve insurance policies. Of note, several payer classes (non‐Medicaid and non‐Medicare), as well as people living in high‐minority population communities, had lower odds of GC; the relationship between these findings and access to care issues should be investigated.

The importance of SDoH in cancer diagnosis and treatment has been shown in systematic reviews^32^, ^33^ and American Cancer Society Blueprint papers.^34^ Its importance in diagnosis and management of GC precursor disorders was further demonstrated by our findings that insurance and lack thereof; unemployment; poverty; lack of education; and living in a high‐minority population neighborhood were associated with increased H. pylori infection risk. Combinations of these variables were also associated with other pre‐GC disorders.

Although patients reported few overall barriers to care, the majority of patients interviewed did not discuss H. pylori with their provider, nor was its risk for GC discussed. Patients also indicated that they would encourage others to seek care sooner. As above, this indicates an opportunity and a need to educate providers and patients on H. pylori symptoms, risks, and treatment.

This study had several limitations. We did not examine H. pylori status as a factor for increased GC risk because the quality of the administrative data was variable. As the most well‐established RF for non‐cardia GC, this was assumed. In addition, as a potential mediator, H. pylori status was not adjusted for because it may suppress associations between RFs and outcomes. Nativity status was also not available; it has been shown to be an important SDoH for Hispanics/Latinos in Texas, related to lack of insurance and SES.^31^ Although primary care providers were interviewed for this study, no patient interviews were conducted with patients in primary care settings due to COVID‐19 limitations. Future studies including this population may point to early barriers to care that may be amenable to intervention.

## 5 CONCLUSIONS

SDoH are important contributors to risk for GC and its precancerous precursors. They should be considered in future studies, especially interventions targeted at improving diagnosis and treatment of these disorders. Specifically, insurance policies should be improved so patients can more easily obtain treatment for *H. pylori*. Providers and patients would benefit from more education on H. pylori symptoms, risks and treatment. Including SDoH in diagnosis and treatment plans for minority patients can play an important role in mitigating the risk for GC and its precursors.

## AUTHOR CONTRIBUTIONS

**Dorothy Long Parma:** Conceptualization (equal); data curation (equal); formal analysis (equal); investigation (equal); methodology (equal); project administration (equal); writing – original draft (equal); writing – review and editing (equal). **Erin P. Finley:** Conceptualization (supporting); formal analysis (supporting); methodology (equal); writing – review and editing (equal). **Roman Fernandez:** Data curation (equal); formal analysis (equal); software (equal). **Jonathan A. L. Gelfond:** Conceptualization (supporting); formal analysis (equal); methodology (equal); software (lead); writing – review and editing (supporting). **Amelie G. Ramirez:** Conceptualization (supporting); funding acquisition (equal); resources (lead); supervision (lead); writing – review and editing (supporting).

## FUNDING INFORMATION

Supported by the U.S. Department of Defense > U.S. Army > U.S. Army Medical Command > Congressionally Directed Medical Research Program > DoD Peer Reviewed Cancer Research Program (W81XWH1910215 to DLP) and the U.S. Department of Health and Human Services > National Institutes of Health > National Cancer Institute—*Redes En Acción*: The National Latino Cancer Research Network (U01 CA86117 and U54 CA153511) and The Cancer Center Support Group of the Mays Cancer Center at The University of Texas Health Science Center at San Antonio (P30 CA054174).

## CONFLICT OF INTEREST STATEMENT

The authors state they have no conflicts of interest to disclose.

## ETHICS STATEMENT

This study was approved by the Institutional Review Board of the University of Texas Health Science Center at San Antonio, 2019‐387E and 2019‐0534H.

## CONSENT

Providers and patients were given an information sheet and provided verbal consent to be interviewed for this study.

## Supporting information


Figure S1.

Figure S2.

Table S1.

Table S2.

## Data Availability

Data is available upon reasonable request.

## References

[1] American Cancer Society . Cancer Facts and figures for Hispanic/Latino Peope 2021–2023. Available from URL: https://www.cancer.org/content/dam/cancer‐org/research/cancer‐facts‐and‐statistics/cancer‐facts‐and‐figures‐for‐hispanics‐and‐latinos/hispanic‐latino‐2021‐2023‐cancer‐facts‐and‐figures.pdf 2021.

[2] Wang Z , Graham DY , Khan A , et al. Incidence of gastric cancer in the USA during 1999 to 2013: a 50‐state analysis. Int J Epidemiol. 2018;47:966‐975. 29672681 10.1093/ije/dyy055 PMC6005108

[3] Miller KD , Goding Sauer A , Ortiz AP , et al. Cancer statistics for Hispanics/Latinos, 2018. CA Cancer J Clin. 2018;68:425‐445. 30285281 10.3322/caac.21494

[4] Li J . Gastric cancer in young adults: a different clinical entity from carcinogenesis to prognosis. Gastroenterol Res Pract. 2020;2020:9512707. 32190044 10.1155/2020/9512707 PMC7071806

[5] Merchant SJ , Kim J , Choi AH , Sun V , Chao J , Nelson R . A rising trend in the incidence of advanced gastric cancer in young Hispanic men. Gastric Cancer. 2017;20:226‐234. 26924751 10.1007/s10120-016-0603-7 PMC5630456

[6] Holowatyj AN , Ulrich CM , Lewis MA . Racial/ethnic patterns of young‐onset noncardia gastric cancer. Cancer Prev Res (Phila). 2019;12:771‐780. 31420363 10.1158/1940-6207.CAPR-19-0200 PMC6825562

[7] Pinheiro PS , Callahan KE , Siegel RL , et al. Cancer mortality in Hispanic ethnic groups. Cancer Epidemiol Biomarkers Prev. 2017;26:376‐382. 28223429 10.1158/1055-9965.EPI-16-0684 PMC5336466

[8] Casamayor M , Morlock R , Maeda H , Ajani J . Targeted literature review of the global burden of gastric cancer. Ecancermedicalscience. 2018;12:883. 30679950 10.3332/ecancer.2018.883 PMC6345079

[9] Corral JE , Delgado Hurtado JJ , Dominguez RL , Valdez de Cuellar M , Balmore Cruz C , Morgan DR . The descriptive epidemiology of gastric cancer in Central America and comparison with United States Hispanic populations. J Gastrointest Cancer. 2015;46:21‐28. 25412859 10.1007/s12029-014-9672-1 PMC4503236

[10] Helicobacter and Cancer Collaborative Group . Gastric cancer and *Helicobacter pylori*: a combined analysis of 12 case control studies nested within prospective cohorts. Gut. 2001;49:347‐353. 11511555 10.1136/gut.49.3.347 PMC1728434

[11] Chen XZ , Schottker B , Castro FA , et al. Association of *Helicobacter pylori* infection and chronic atrophic gastritis with risk of colonic, pancreatic and gastric cancer: a ten‐year follow‐up of the ESTHER cohort study. Oncotarget. 2016;7:17182‐17193. 26958813 10.18632/oncotarget.7946 PMC4941379

[12] Gonzalez CA , Megraud F , Buissonniere A , et al. *Helicobacter pylori* infection assessed by ELISA and by immunoblot and noncardia gastric cancer risk in a prospective study: the Eurogast‐EPIC project. Ann Oncol. 2012;23:1320‐1324. 21917738 10.1093/annonc/mdr384

[13] Malfertheiner P , Megraud F , O'Morain CA , et al. Management of *Helicobacter pylori* infection‐the Maastricht V/Florence consensus report. Gut. 2017;66:6‐30. 27707777 10.1136/gutjnl-2016-312288

[14] Moss SF . The clinical evidence linking *Helicobacter pylori* to gastric cancer. Cell Mol Gastroenterol Hepatol. 2016;3:183‐191. 28275685 10.1016/j.jcmgh.2016.12.001 PMC5331857

[15] Choi CE , Sonnenberg A , Turner K , Genta RM . High prevalence of gastric preneoplastic lesions in east Asians and Hispanics in the USA. Dig Dis Sci. 2015;60:2070‐2076. 25724165 10.1007/s10620-015-3591-2

[16] Schlansky B , Sonnenberg A . Epidemiology of noncardia gastric adenocarcinoma in the United States. Am J Gastroenterol. 2011;106:1978‐1985. 22008896 10.1038/ajg.2011.213

[17] Chey WD , Leontiadis GI , Howden CW , Moss SF . ACG clinical guideline: treatment of *Helicobacter pylori* infection. Am J Gastroenterol. 2017;112:212‐239. 28071659 10.1038/ajg.2016.563

[18] Texas Department of State Health Services . Health Facts Profile (2014–2015). Accessed March 22, 2021. http://healthdata.dshs.texas.gov/dashboard/surveys‐and‐profiles/health‐facts‐profile‐2014‐2015

[19] U.S. Census Bureau . State and County QuickFacts. Data derived from Population Estimates, American Community Survey, Census of Population and Housing, State and County Housing Unit Estimates, County Business Patterns, Nonemployer Statistics, Economic Census, Survey of Business Owners, Building Permits. Accessed March 22, 2021. https://www.census.gov/quickfacts/fact/table/US,TX,starrcountytexas,willacycountytexas,brookscountytexas/PST045218

[20] Archer N , Gallion KJ , Holden AEC , Long Parma D , Munoz E , Suarez L . The South Texas Health Status Review: A Health Disparities Roadmap. 2nd ed. Springer Science; 2013. 31314218

[21] Gupta S , Tao L , Murphy JD , et al. Race/ethnicity‐, socioeconomic status‐, and anatomic subsite‐specific risks for gastric cancer. Gastroenterology. 2019;156:59‐62.e54. 30267713 10.1053/j.gastro.2018.09.045 PMC6309455

[22] Fetters MD , Curry LA , Creswell JW . Achieving integration in mixed methods designs‐principles and practices. Health Serv Res. 2013;48:2134‐2156. 24279835 10.1111/1475-6773.12117 PMC4097839

[23] Regenstrief Institute . About LOINC. Accessed May 16, 2018. https://loinc.org/about/ 2018.

[24] U.S. Census Bureau . State and County QuickFacts. Data derived from Population Estimates, American Community Survey, Census of Population and Housing, State and County Housing Unit Estimates, County Business Patterns, Nonemployer Statistics, Economic Census, Survey of Business Owners, Building Permits. Accessed September 25, 2017. https://www.census.gov/quickfacts/fact/map/TX/RHI225216#viewtop

[25] Feldstein AC , Glasgow RE . A practical, robust implementation and sustainability model (PRISM) for integrating research findings into practice. Jt Comm J Qual Patient Saf. 2008;34:228‐243. 18468362 10.1016/s1553-7250(08)34030-6

[26] Finley EP , Schneegans S , Tami C , et al. Implementing prescription drug monitoring and other clinical decision support for opioid risk mitigation in a military health care setting: a qualitative feasibility study. J Am Med Inform Assoc. 2018;25:515‐522. 29025024 10.1093/jamia/ocx075 PMC7646964

[27] Abraham TH , Finley EP , Drummond KL , et al. A method for developing trustworthiness and preserving richness of qualitative data during team‐based analysis of large data sets. Am J Eval. 2021;42:139‐156.

[28] Tong A , Sainsbury P , Craig J . Consolidated criteria for reporting qualitative research (COREQ): a 32‐item checklist for interviews and focus groups. Int J Qual Health Care. 2007;19:349‐357. 17872937 10.1093/intqhc/mzm042

[29] Grad YH , Lipsitch M , Aiello AE . Secular trends in *Helicobacter pylori* seroprevalence in adults in the United States: evidence for sustained race/ethnic disparities. Am J Epidemiol. 2012;175:54‐59. 22085628 10.1093/aje/kwr288 PMC3244610

[30] Patterson T , Straten E , Jimenez S . The prevalence of *Helicobacter pylori* antibody in different age groups in Central Texas. Clin Lab Sci. 2012;25:102‐106. 22693779

[31] Ju MR , Karalis JD , Bhat A , et al. Nativity status is an important social determinant of health for Hispanic patients with gastric cancer in Texas. Ann Surg Oncol. 2022;29:3113‐3121. 35028796 10.1245/s10434-021-11188-3

[32] Korn AR , Walsh‐Bailey C , Correa‐Mendez M , et al. Social determinants of health and US cancer screening interventions: a systematic review. CA Cancer J Clin. 2023;73:461‐479. 37329257 10.3322/caac.21801 PMC10529377

[33] Mohan G , Chattopadhyay S . Cost‐effectiveness of leveraging social determinants of health to improve breast, cervical, and colorectal cancer screening: a systematic review. JAMA Oncol. 2020;6:1434‐1444. 32556187 10.1001/jamaoncol.2020.1460 PMC7857975

[34] Alcaraz KI , Wiedt TL , Daniels EC , Yabroff KR , Guerra CE , Wender RC . Understanding and addressing social determinants to advance cancer health equity in the United States: a blueprint for practice, research, and policy. CA Cancer J Clin. 2020;70:31‐46. 31661164 10.3322/caac.21586

