# Supplementary figures and images for: Facilitators of and barriers to gastric cancer and precursor diagnosis among South Texas residents: Social determinants of health
Source: Cancer Med. 2024 Mar 20;13(6):e7002. doi: 10.1002/cam4.7002 (PMC10952019; doi:10.1002/cam4.7002)

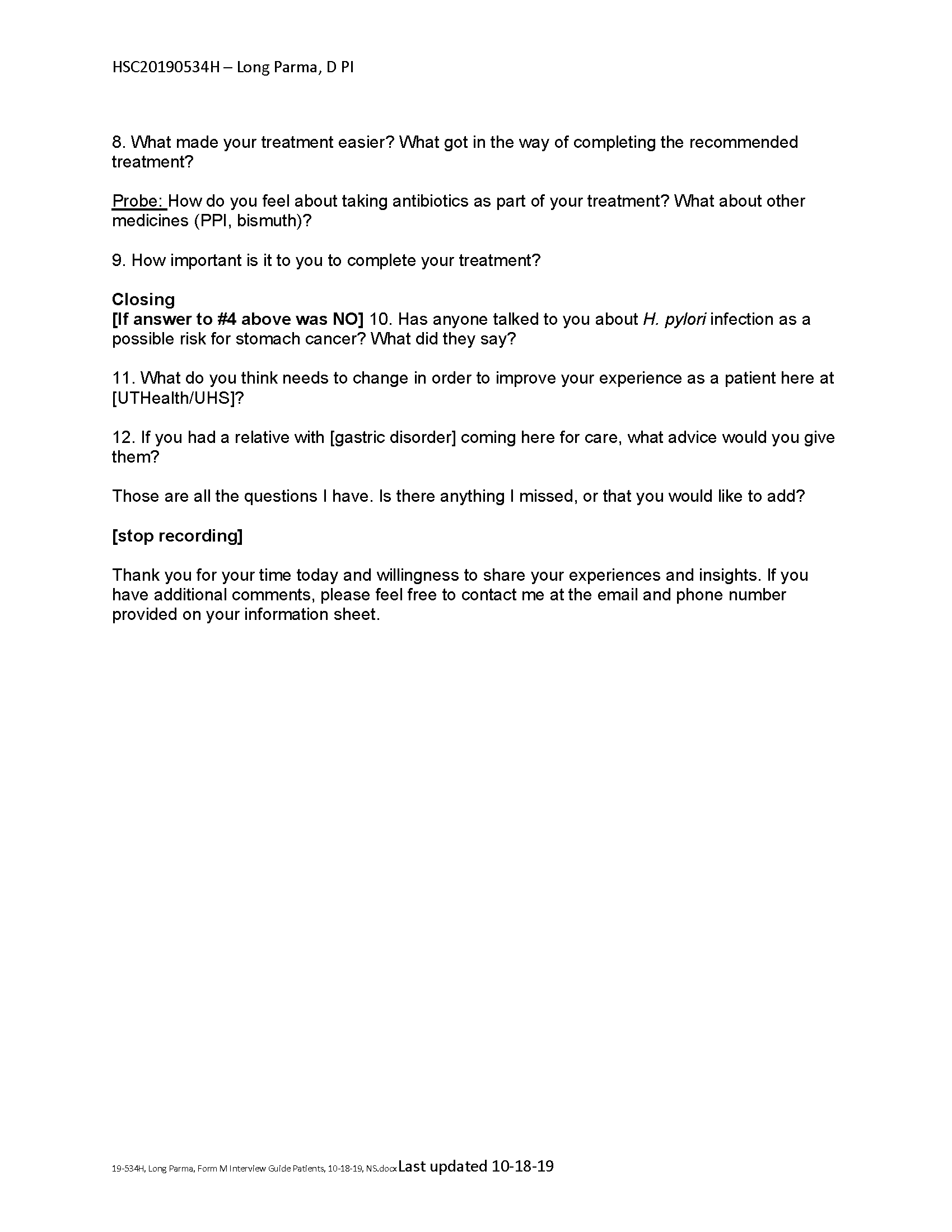

Supplement: Supplementary file 1 — Figure S1. [file CAM4-13-e7002-s002.zip › cam47002-sup-0002-FigureS2.tif]

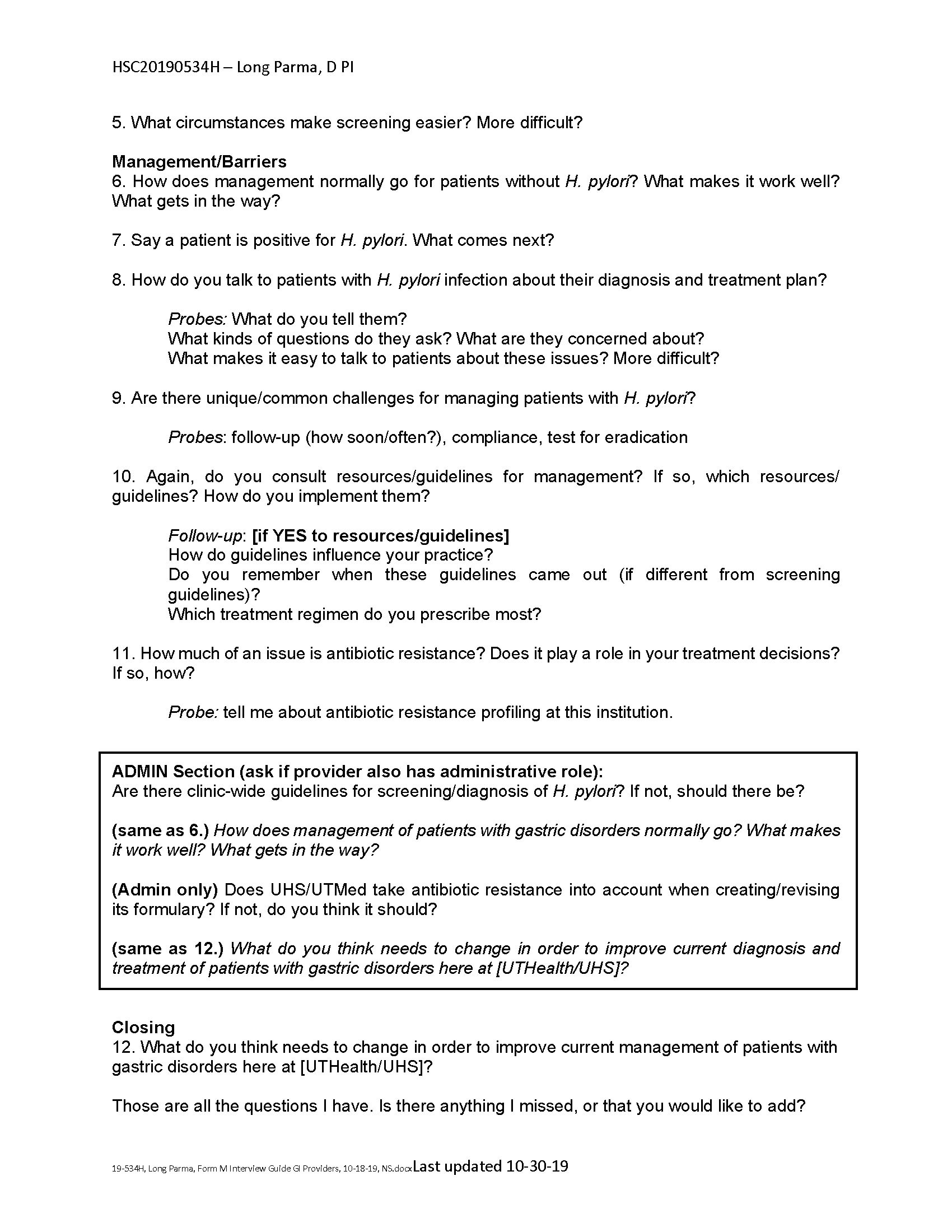

Supplement: Supplementary file 2 — Figure S2. [file CAM4-13-e7002-s003.zip › cam47002-sup-0004-FigureS4.tiff]

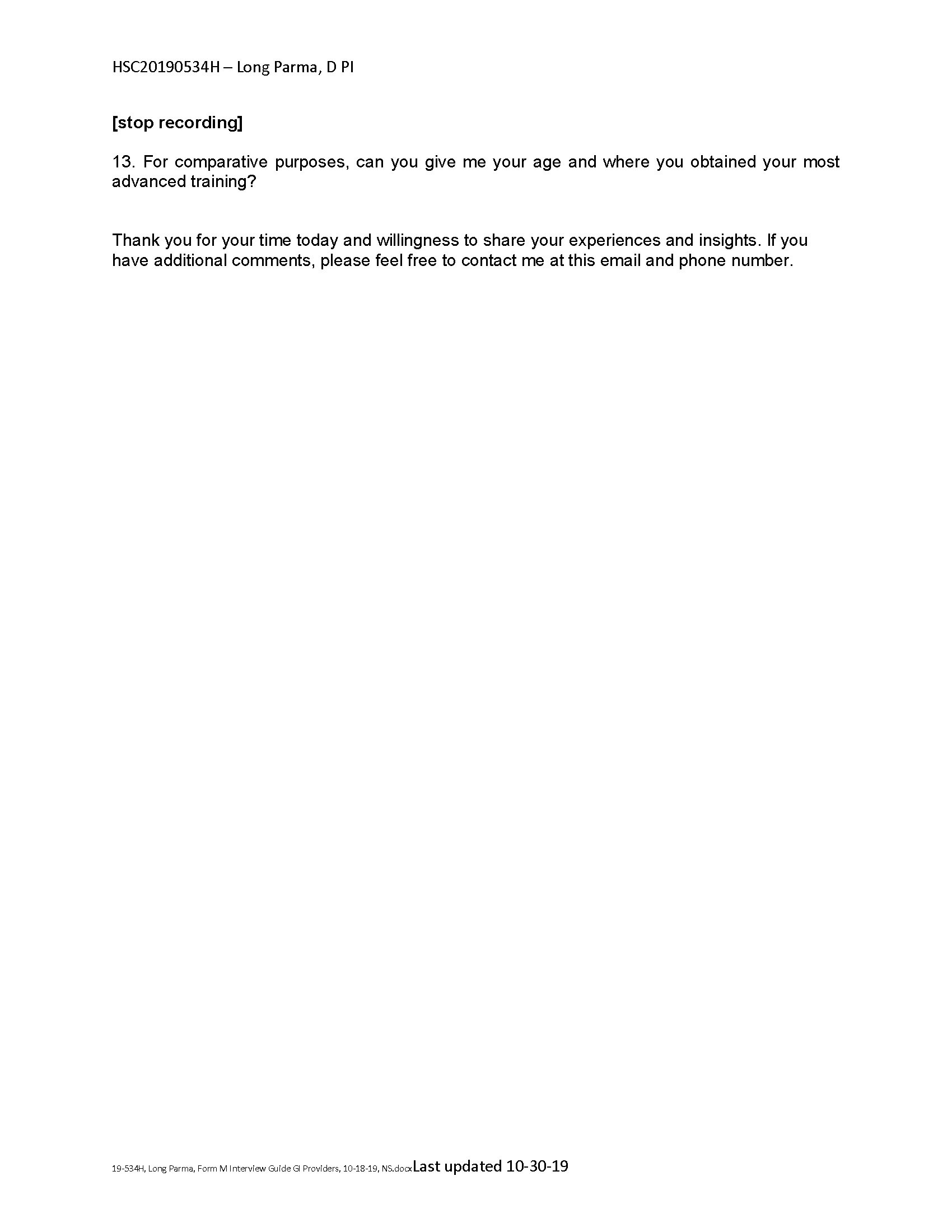

Supplement: Supplementary file 2 — Figure S2. [file CAM4-13-e7002-s003.zip › cam47002-sup-0005-FigureS2.tiff]
