# Supplementary material for: Facilitators of and barriers to gastric cancer and precursor diagnosis among South Texas residents: Social determinants of health
Source: Cancer Med. 2024 Mar 20;13(6):e7002. doi: 10.1002/cam4.7002 (PMC10952019; doi:10.1002/cam4.7002)
Supplement: Supplementary file 3 — Table S1. [file CAM4-13-e7002-s004.docx]

| **Characteristic** | Atrophic.gastritis | | | Gastric.ulcer | | | Gastritis | | | H.pylori | | | Intestinal.metaplasia | | | Other | | |
| --- | --- | --- | --- | --- | --- | --- | --- | --- | --- | --- | --- | --- | --- | --- | --- | --- | --- | --- |
|  | **OR***1* | **95% CI***1* | **p-value** | **OR***1* | **95% CI***1* | **p-value** | **OR***1* | **95% CI***1* | **p-value** | **OR***1* | **95% CI***1* | **p-value** | **OR***1* | **95% CI***1* | **p-value** | **OR***1* | **95% CI***1* | **p-value** |
| Race_Ethnicity |  |  |  |  |  |  |  |  |  |  |  |  |  |  |  |  |  |  |
| White | — | — |  | — | — |  | — | — |  | — | — |  | — | — |  | — | — |  |
| Black | 1.04 | 0.85, 1.28 | 0.7 | 1.18 | 0.99, 1.40 | 0.056 | 1 | 0.85, 1.18 | >0.9 | 1.36 | 1.19, 1.54 | <0.001 | 0.92 | 0.38, 1.94 | 0.8 | 1.18 | 0.83, 1.66 | 0.3 |
| Hispanic | 1.1 | 0.99, 1.22 | 0.09 | 0.9 | 0.82, 0.99 | 0.037 | 1.18 | 1.08, 1.28 | <0.001 | 1.41 | 1.32, 1.51 | <0.001 | 0.87 | 0.58, 1.32 | 0.5 | 1.26 | 1.05, 1.51 | 0.016 |
| Other | 1.07 | 0.91, 1.25 | 0.4 | 0.77 | 0.66, 0.89 | <0.001 | 0.91 | 0.80, 1.03 | 0.14 | 1.44 | 1.31, 1.59 | <0.001 | 1.59 | 0.94, 2.65 | 0.078 | 1.05 | 0.79, 1.39 | 0.7 |
| Payer.Class.Revised |  |  |  |  |  |  |  |  |  |  |  |  |  |  |  |  |  |  |
| Private | — | — |  | — | — |  | — | — |  | — | — |  | — | — |  | — | — |  |
| Carelink | 1.13 | 0.99, 1.29 | 0.072 | 0.47 | 0.41, 0.53 | <0.001 | 0.61 | 0.55, 0.67 | <0.001 | 2.05 | 1.88, 2.24 | <0.001 | 0.02 | 0.00, 0.07 | <0.001 | 0.64 | 0.51, 0.80 | <0.001 |
| Government | 0.99 | 0.81, 1.22 | >0.9 | 0.75 | 0.62, 0.90 | 0.002 | 0.96 | 0.84, 1.11 | 0.6 | 1.12 | 0.98, 1.28 | 0.1 | 1.11 | 0.62, 1.91 | 0.7 | 1.35 | 1.00, 1.82 | 0.05 |
| Medicaid | 1.18 | 0.95, 1.45 | 0.12 | 1.18 | 0.99, 1.40 | 0.062 | 0.78 | 0.66, 0.91 | 0.002 | 1.4 | 1.22, 1.61 | <0.001 | 0.14 | 0.02, 0.45 | 0.006 | 0.82 | 0.56, 1.17 | 0.3 |
| Medicare | 0.86 | 0.72, 1.02 | 0.085 | 0.88 | 0.76, 1.01 | 0.074 | 0.86 | 0.74, 0.99 | 0.038 | 1 | 0.88, 1.13 | >0.9 | 0.66 | 0.40, 1.07 | 0.092 | 0.93 | 0.71, 1.21 | 0.6 |
| Medicare + Medicaid | 0.29 | 0.07, 0.78 | 0.037 | 2.75 | 1.66, 4.54 | <0.001 | 0.84 | 0.43, 1.52 | 0.6 | 0.48 | 0.24, 0.89 | 0.028 | 0.66 | 0.04, 3.17 | 0.7 | 0.79 | 0.19, 2.17 | 0.7 |
| Military | 0.51 | 0.18, 1.15 | 0.15 | 1.01 | 0.55, 1.74 | >0.9 | 0.79 | 0.45, 1.32 | 0.4 | 0.88 | 0.53, 1.40 | 0.6 | 0.86 | 0.05, 4.01 | 0.9 | 1.14 | 0.34, 2.81 | 0.8 |
| Missing | 0.52 | 0.38, 0.70 | <0.001 | 0.52 | 0.41, 0.66 | <0.001 | 0.45 | 0.36, 0.56 | <0.001 | 1.95 | 1.68, 2.28 | <0.001 | 1.69 | 0.98, 2.83 | 0.051 | 1.26 | 0.88, 1.78 | 0.2 |
| Other | 0.83 | 0.63, 1.07 | 0.2 | 0.4 | 0.30, 0.52 | <0.001 | 0.29 | 0.22, 0.37 | <0.001 | 1.96 | 1.68, 2.29 | <0.001 | 0.1 | 0.01, 0.44 | 0.021 | 0.43 | 0.24, 0.73 | 0.003 |
| Self-Pay | 0.48 | 0.37, 0.62 | <0.001 | 1.17 | 0.99, 1.38 | 0.062 | 1.76 | 1.54, 2.01 | <0.001 | 1.19 | 1.04, 1.36 | 0.01 | 0 | 0.00, 0.20 | >0.9 | 0.55 | 0.36, 0.81 | 0.004 |
| MINORITYPOP_2016_5Y_QUARTILE |  |  |  |  |  |  |  |  |  |  |  |  |  |  |  |  |  |  |
| **Characteristic** | Atrophic.gastritis | | | Gastric.ulcer | | | Gastritis | | | H.pylori | | | Intestinal.metaplasia | | |  |  |  |
|  | **OR***1* | **95% CI***1* | **p-value** | **OR***1* | **95% CI***1* | **p-value** | **OR***1* | **95% CI***1* | **p-value** | **OR***1* | **95% CI***1* | **p-value** | **OR***1* | **95% CI***1* | **p-value** |  |  |  |
| Q1 | — | — |  | — | — |  | — | — |  | — | — |  | — | — |  | — | — |  |
| Q2 | 1.04 | 0.90, 1.20 | 0.6 | 1.02 | 0.90, 1.16 | 0.7 | 1.09 | 0.98, 1.21 | 0.12 | 1.35 | 1.24, 1.48 | <0.001 | 0.54 | 0.32, 0.88 | 0.015 | 0.75 | 0.59, 0.95 | 0.02 |
| Q3 | 1.07 | 0.93, 1.24 | 0.3 | 0.89 | 0.78, 1.01 | 0.068 | 1.1 | 0.98, 1.22 | 0.1 | 1.43 | 1.31, 1.56 | <0.001 | 0.59 | 0.36, 0.95 | 0.033 | 0.93 | 0.74, 1.17 | 0.5 |
| Q4 | 1.22 | 1.07, 1.38 | 0.002 | 0.87 | 0.78, 0.98 | 0.018 | 1.08 | 0.98, 1.19 | 0.12 | 1.67 | 1.54, 1.81 | <0.001 | 0.28 | 0.16, 0.46 | <0.001 | 0.88 | 0.71, 1.08 | 0.2 |
| NOHS_2016_5Y_QUARTILE |  |  |  |  |  |  |  |  |  |  |  |  |  |  |  |  |  |  |
| Q1 | — | — |  | — | — |  | — | — |  | — | — |  | — | — |  | — | — |  |
| Q2 | 1.09 | 0.94, 1.28 | 0.3 | 0.99 | 0.87, 1.13 | 0.9 | 0.94 | 0.84, 1.05 | 0.3 | 1.26 | 1.14, 1.38 | <0.001 | 0.53 | 0.35, 0.82 | 0.004 | 0.83 | 0.66, 1.06 | 0.14 |
| Q3 | 1.29 | 1.10, 1.51 | 0.002 | 0.94 | 0.82, 1.08 | 0.4 | 0.98 | 0.87, 1.10 | 0.7 | 1.5 | 1.36, 1.66 | <0.001 | 0.24 | 0.13, 0.43 | <0.001 | 0.81 | 0.63, 1.05 | 0.11 |
| Q4 | 1.25 | 1.07, 1.47 | 0.006 | 0.96 | 0.83, 1.10 | 0.5 | 0.95 | 0.85, 1.07 | 0.4 | 1.7 | 1.54, 1.88 | <0.001 | 0.27 | 0.15, 0.46 | <0.001 | 0.89 | 0.69, 1.15 | 0.4 |
| MEDINCOME_2016_5Y_QUARTILE |  |  |  |  |  |  |  |  |  |  |  |  |  |  |  |  |  |  |
| Q1 | — | — |  | — | — |  | — | — |  | — | — |  | — | — |  | — | — |  |
| Q2 | 0.94 | 0.82, 1.08 | 0.4 | 0.98 | 0.87, 1.12 | 0.8 | 0.99 | 0.89, 1.10 | 0.8 | 0.97 | 0.89, 1.05 | 0.4 | 1.1 | 0.51, 2.38 | 0.8 | 1 | 0.79, 1.27 | >0.9 |
| Q3 | 0.88 | 0.77, 1.01 | 0.066 | 1.18 | 1.05, 1.33 | 0.006 | 0.97 | 0.88, 1.08 | 0.6 | 0.83 | 0.76, 0.90 | <0.001 | 2.01 | 1.07, 3.99 | 0.036 | 0.95 | 0.75, 1.20 | 0.7 |
| Q4 | 0.86 | 0.76, 0.99 | 0.029 | 1.08 | 0.95, 1.22 | 0.2 | 0.98 | 0.89, 1.09 | 0.8 | 0.67 | 0.62, 0.73 | <0.001 | 4.14 | 2.35, 7.89 | <0.001 | 1.11 | 0.89, 1.39 | 0.3 |
| UNEMPLOYED_2016_5Y_QUARTILE |  |  |  |  |  |  |  |  |  |  |  |  |  |  |  |  |  |  |
| Q1 | — | — |  | — | — |  | — | — |  | — | — |  | — | — |  | — | — |  |
| Q2 | 1 | 0.87, 1.15 | >0.9 | 0.86 | 0.76, 0.98 | 0.021 | 0.99 | 0.89, 1.10 | 0.8 | 1 | 0.91, 1.08 | >0.9 | 1.07 | 0.68, 1.72 | 0.8 | 1.05 | 0.83, 1.32 | 0.7 |
| **Characteristic** | Atrophic.gastritis | | | Gastric.ulcer | | | Gastritis | | | H.pylori | | | Intestinal.metaplasia | | |  |  |  |
|  | **OR***1* | **95% CI***1* | **p-value** | **OR***1* | **95% CI***1* | **p-value** | **OR***1* | **95% CI***1* | **p-value** | **OR***1* | **95% CI***1* | **p-value** | **OR***1* | **95% CI***1* | **p-value** |  |  |  |
| Q3 | 1.04 | 0.91, 1.20 | 0.5 | 0.96 | 0.85, 1.09 | 0.5 | 0.99 | 0.89, 1.10 | 0.9 | 1.12 | 1.03, 1.22 | 0.011 | 0.81 | 0.48, 1.34 | 0.4 | 1.07 | 0.84, 1.36 | 0.6 |
| Q4 | 1.09 | 0.96, 1.24 | 0.2 | 0.95 | 0.85, 1.07 | 0.4 | 0.97 | 0.88, 1.07 | 0.5 | 1.18 | 1.09, 1.28 | <0.001 | 0.5 | 0.28, 0.86 | 0.013 | 1.06 | 0.84, 1.33 | 0.6 |
| UNINSURED_2016_5Y_QUARTILE |  |  |  |  |  |  |  |  |  |  |  |  |  |  |  |  |  |  |
| Q1 | — | — |  | — | — |  | — | — |  | — | — |  | — | — |  | — | — |  |
| Q2 | 1.03 | 0.88, 1.19 | 0.7 | 1.07 | 0.94, 1.22 | 0.3 | 1.02 | 0.92, 1.14 | 0.7 | 1.27 | 1.16, 1.40 | <0.001 | 0.57 | 0.34, 0.92 | 0.024 | 0.78 | 0.60, 1.00 | 0.047 |
| Q3 | 1.16 | 1.01, 1.33 | 0.04 | 1.09 | 0.96, 1.24 | 0.2 | 1.01 | 0.91, 1.12 | 0.9 | 1.28 | 1.18, 1.40 | <0.001 | 0.44 | 0.27, 0.72 | 0.001 | 0.89 | 0.71, 1.12 | 0.3 |
| Q4 | 1.16 | 1.02, 1.32 | 0.023 | 0.99 | 0.88, 1.11 | 0.9 | 0.98 | 0.89, 1.08 | 0.7 | 1.52 | 1.40, 1.64 | <0.001 | 0.31 | 0.19, 0.50 | <0.001 | 0.83 | 0.68, 1.03 | 0.085 |
| POOR_2016_5Y_QUARTILE |  |  |  |  |  |  |  |  |  |  |  |  |  |  |  |  |  |  |
| Q1 | — | — |  | — | — |  | — | — |  | — | — |  | — | — |  | — | — |  |
| Q2 | 0.94 | 0.80, 1.11 | 0.5 | 1.01 | 0.87, 1.16 | >0.9 | 1.09 | 0.96, 1.23 | 0.2 | 1.18 | 1.07, 1.31 | 0.002 | 0.74 | 0.47, 1.19 | 0.2 | 0.76 | 0.59, 0.99 | 0.042 |
| Q3 | 1.07 | 0.92, 1.25 | 0.4 | 1.02 | 0.89, 1.18 | 0.8 | 1.06 | 0.94, 1.20 | 0.4 | 1.38 | 1.25, 1.53 | <0.001 | 0.36 | 0.22, 0.62 | <0.001 | 0.79 | 0.62, 1.01 | 0.059 |
| Q4 | 1.07 | 0.91, 1.26 | 0.4 | 0.96 | 0.83, 1.11 | 0.6 | 1.04 | 0.92, 1.18 | 0.6 | 1.49 | 1.34, 1.65 | <0.001 | 0.26 | 0.14, 0.47 | <0.001 | 0.8 | 0.62, 1.04 | 0.095 |
